# Using body mass index to estimate individualised patient radiation dose in abdominal computed tomography

**DOI:** 10.1186/s41747-018-0070-5

**Published:** 2018-11-28

**Authors:** Siobhan O’Neill, Richard G. Kavanagh, Brian W. Carey, Niamh Moore, Michael Maher, Owen J. O’Connor

**Affiliations:** 1 Department of Radiology, Cork University Hospital, Wilton, Cork, Ireland; 2 Department of Radiology, University College Cork, Cork, Ireland; 3 APC Microbiome Ireland, University College Cork, Cork, Ireland

**Keywords:** Abdomen, Body mass index, Tomography (x-ray, computed), Radiation dosage

## Abstract

**Background:**

The size-specific dose estimate (SSDE) is a dose-related metrics that incorporates patient size into its calculation. It is usually derived from the volume computed tomography dose index (CTDI_vol_) by applying a conversion factor determined from manually measured anteroposterior and lateral skin-to-skin patient diameters at the midslice level on computed tomography (CT) localiser images, an awkward, time-consuming, and not highly reproducible technique. The objective of this study was to evaluate the potential for the use of body mass index (BMI) as a size-related metrics alternative to the midslice effective diameter (*D*_*E*_) to obtain a size-specific dose (SSDE) in abdominal CT.

**Methods:**

In this retrospective study of patients who underwent abdominal CT for the investigation of inflammatory bowel disease, the *D*_*E*_ was measured on the midslice level on CT-localiser images of each patient. This was correlated with patient BMI and the linear regression equation relating the quantities was calculated. The ratio between the internal and the external abdominal diameters (*D*_RATIO_) was also measured to assess correlation with radiation dose. Pearson correlation analysis and linear regression models were used.

**Results:**

There was good correlation between *D*_*E*_ and patient BMI (*r* = 0.88). An equation allowing calculation of *D*_*E*_ from BMI was calculated by linear regression analysis as follows: *D*_*E*_ = 0.76 (*BMI*) + 9.4. A weak correlation between radiation dose and *D*_RATIO_ was demonstrated (*r* = 0.45).

**Conclusions:**

Patient BMI can be used to accurately estimate *D*_*E*_, obviating the need to measure anteroposterior and lateral diameters in order to calculate a SSDE for abdominal CT.

## Key points

- A strong correlation between BMI and *D*_*E*_ was found
- BMI can be used to estimate patient *D*_*E*_
- Calculating patient BMI can facilitate individualised patient radiation dose estimation in abdominal CT

## Background

The individual patient radiation dose from computed tomography (CT) is notoriously difficult to estimate. There is a growing interest in this topic, however, due to the ever-increasing CT use and general concerns about the risks associated with radiation exposure from medical imaging. This has precipitated the increased use of dose monitoring systems in clinical practice.

Current CT scanner radiation dose output following patient imaging is displayed in the CT dose report in terms of volume CT dose index (CTDI_vol_) and dose length product (DLP), standardised measurements deduced from homogenous phantoms under normalised conditions [1, 2]. These parameters do not provide a direct measure of the individualised patient radiation dose, a variable that is dependent on patient size.

The size-specific dose estimate (SSDE) is a dose-related metrics that incorporates patient size into its calculation. This metric has been advocated for the reporting of patient radiation dose in CT by the American Association of Physicists in Medicine (AAPM) task group 204 and has increasingly been applied and accepted [3, 4]. The SSDE is derived from the CTDI_vol_ by applying a conversion factor determined from manually measured anteroposterior and lateral skin-to-skin patient diameters at the midslice level on CT-localiser images [3]. On a practical level this technique can be awkward and time-consuming, and open to interobserver measurement variability.

The objective of this study was to evaluate the potential for using body mass index (BMI) as an alternative size metrics, in lieu of measured body diameters, to estimate patient effective diameter (*D*_*E*_). Thus, estimation of SSDE at the time of CT scanning would be more user-friendly, contributing positively to patient radiation dose optimisation.

## Methods

### Subjects

This study was performed retrospectively on CT data acquired as part of a clinical trial protocol investigating the use of CT in inflammatory bowel disease (ClinicalTrials.gov Identifier: NCT 01244386) [5] with approval from the institution Clinical Research Ethics Committee. Fifty adult patients were included in this study and, as part of this trial protocol, all patients signed informed consent.

### CT scan protocol

All patients underwent a CT scan of the abdomen and pelvis with a standardised protocol using the following parameters: scan range encompassing the lung bases to the pubic symphysis; 0.625-mm slice acquisition thickness; intravenously administered contrast (Iohexol, Omnipaque 300, General Electric Healthcare, Waukesha, WI, USA) delivered at 2.5 mL/s and imaged in the portal venous phase; 1.5 L of positive oral contrast (2% Gastrografin, Bracco Diagnostics Inc., Princeton, NJ, USA); tube voltage of 120 kVp; automated tube current modulation resulting in a variable current with a minimum of 50 mA and a maximum of 350 mA; gantry rotation time of 0.8 s; noise index 38. All CT images were acquired using a single 64-slice multi-detector row CT scanner (Lightspeed VCT-XTe, GE Healthcare, General Electric Medical Systems, Waukesha, WI, USA). The DLP and CTDI_vol_ values, as well as the corresponding phantom size, were recorded from each CT dose report. CTDI_vol_ and DLP tolerances were verified using a standard 32-cm Perspex phantom, a 10-cm ionisation chamber with a Victoreen NERO mAx unit (Fluke Biomedical, Solon, OH, USA).

The SSDEs were calculated by multiplying the CTDI_vol_ of each patient by conversion factors corresponding to the effective patient diameters in the AAPM reference tables [3]. The imaging performance and assessment from CT patient dosimetry calculator (ImPACT version 0.99x, London, UK) was used to calculate the effective dose.

### BMI measurement

Each patient had weight and height measurements performed and their BMI calculated immediately prior to CT scan using a dedicated calibrated measuring device (electronic measuring station Model 763, Seca Medical, Hamburg, Germany). BMI data were used to subdivide patient groups, where underweight referred to BMI < 18.5 kg/m^2^, normal weight referred to 18.5 ≤ BMI < 25 kg/m^2^, overweight referred to 25 ≤ BMI < 30 kg/m^2^ and obese referred to BMI ≥ 30 kg/m^2^.

### Body diameter measurements

Images were reviewed on a picture-archiving and communication system (PACS) workstation (Impax 6.3.1, AGFA Healthcare, Morstel, Belgium) in a DICOM format. As per AAPM Report 204 guidelines, body diameters were measured at the midslice level (median image of the craniocaudal scanning length) on the CT-localiser images because, for larger patients, maximum skin-to-skin distance is often not included on transverse CT images [3, 6]. Diameter measurements were performed manually with the electronic callipers available on the PACS using a window width of 350 Hounsfield Units (HU) and window level of 50 HU. From personal experience at our institution, analysis of interoperator variability for PACS-based anthropometric measurements shows no statistically significant differences. Therefore, a single investigator carried out all measurements. A fixed window level and setting was used for each individual study.

**Fig. 1:**
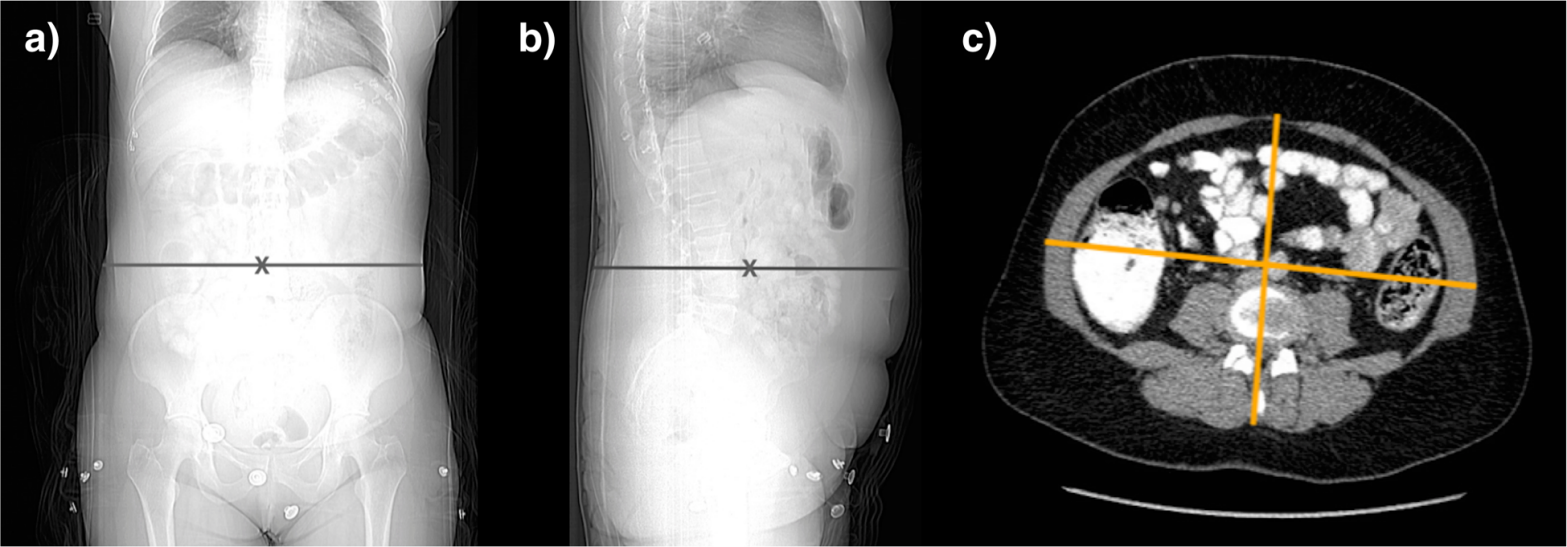
Measurement of lateral (*D*_*LAT*_) (**a**) anteroposterior (*D*_*AP*_) (**b**) skin-to-skin patient diameters at the midslice level on CT-localiser images. Measurement of the inner lateral and anteroposterior diameters on the axial midslice CT image excluding the subcutaneous adipose tissue (**c**) to allow calculation of the effect inner diameter (*D*_*IN*_) and the effective diameter ratio (*D*_*RATIO*_)

Maximum skin-to-skin anteroposterior diameter (*D*_*AP*_) and lateral diameter (*D*_*LAT*_) were measured in centimetres on lateral and anteroposterior localiser images, respectively. *D*_*AP*_ is defined as the anteroposterior skin-to-skin diameter on the lateral localiser at the midslice level (Fig. 1a) while *D*_*LAT*_ is defined as the lateral skin-to-skin diameter on the anteroposterior localiser image at the midslice level (Fig. 1b). The *D*_*E*_ is defined as the diameter of the circle with area equivalent to the cross-sectional area of the patient at the particular z-axis level (i.e. the midslice level) and is calculated as the geometric mean of *D*_*AP*_ and *D*_*LAT*_, as follows:

$$ {D}_E=\surd \left({D}_{AP}\times {D}_{LAT}\right) $$

The *outer D*_*E*_ (*D*_*OUT*_) equates to the conventional *D*_*E*_ calculated using the AAPM method described above. The *inner D*_*E*_ (*D*_*IN*_) is derived using the anteroposterior (*D*_*AP(IN)*_) and lateral diameters (*D*_*LAT(IN)*_) measured on an axial CT image at the midslice level, excluding the subcutaneous adipose tissue (Fig. 1c). The *D*_*IN*_ is then calculated as the geometric mean of *D*_*AP(IN)*_ and *D*_*LAT(IN)*_, as follows:

$$ {D}_{IN}=\surd \left({D}_{AP(IN)}\times {D}_{LAT(IN)}\right) $$

The *D*_*E*_ ratio (*D*_*RATIO*_), another patient size-related metrics proposed by Lamoureux et al. [7], was also calculated, as follows:

$$ {D}_{RATIO}={D}_{OUT}/{D}_{IN} $$

### Statistical analysis

Data were collated using Microsoft Excel 2010 (Microsoft Corporation, Redmond, WA, USA) and statistical analyses were conducted by using Microsoft Excel 2010 and GraphPad Prism version 5.0 (GraphPad Software Inc., San Diego, CA, USA). Descriptive statistics including means, standard deviations and ranges were calculated. BMI, dose indices (CTDI_vol_, DLP, SSDE, effective dose) and body diameters (*D*_*AP*_, *D*_*LAT*_, *D*_*AP*+_*D*_*LAT*_, *D*_*E*,_ *D*_*RATIO*_) were recorded for each patient CT examination. The correlations between BMI, dose indices, and body diameter measurements were examined with Pearson correlation analysis (*r*). Linear regression models were used to assess the dependence of CTDI, DLP, SSDE, and effective dose on BMI. Linear regression models were also used to estimate the relationship of effective diameter (independent variable) with BMI (dependent variable). A *p*-value lower than 0.05 was taken to indicate statistical significance.

## Results

### Patient demographics

The study population (*n* = 50) comprised 19 men and 31 women with an age of 37.9 ± 14.4 years (mean ± standard deviation [SD], ranging from 17 to 73 years.

### BMI and dose metrics

The overall BMI was 24.6 ± 4.8 kg/m^2^ (mean ± SD), ranging from 17.4 to 38.8 kg/m^2^. The mean CTDI_vol_, DLP, SSDE and effective dose measurements overall, and when stratified for BMI, are listed in Table 1.

**Table 1:** Summary of mean values for computed tomography dose metrics, overall and stratified for body mass index (BMI)

	All (*n* = 50)	BMI < 25 kg/m^2^ (*n* = 32)	BMI ≥ 25 kg/m^2^ (*n* = 18)	*p*-value
CTDI_vol_ (mGy)	6.26 ± 3.83	4.33 ± 0.83	9.68 ± 4.65	0.000^a^
DLP (mGy.cm)	299.42 ± 196.06	202.36 ± 41.27	471.96 ± 241.73	0.000^a^
SSDE (mGy)	7.81 ± 3.08	6.22 ± 0.75	10.64 ± 3.61	0.000^a^
Effective dose (mSv)	4.77 ± 3.23	3.18 ± 0.62	7.59 ± 1.64	0.000^a^

*CTDI*_*vol*_ volume-computed tomography dose index, *DLP* dose length product, *SSDE* size-specific dose estimate, *BMI* Body mass index

Data as means ± standard deviations of the mean

^a^Value shows a statistically significant difference with a two-tailed *p*-value of less than 0.05, when the radiation doses of each protocol are compared with one another

### Body diameter measurements

**Fig. 2:**
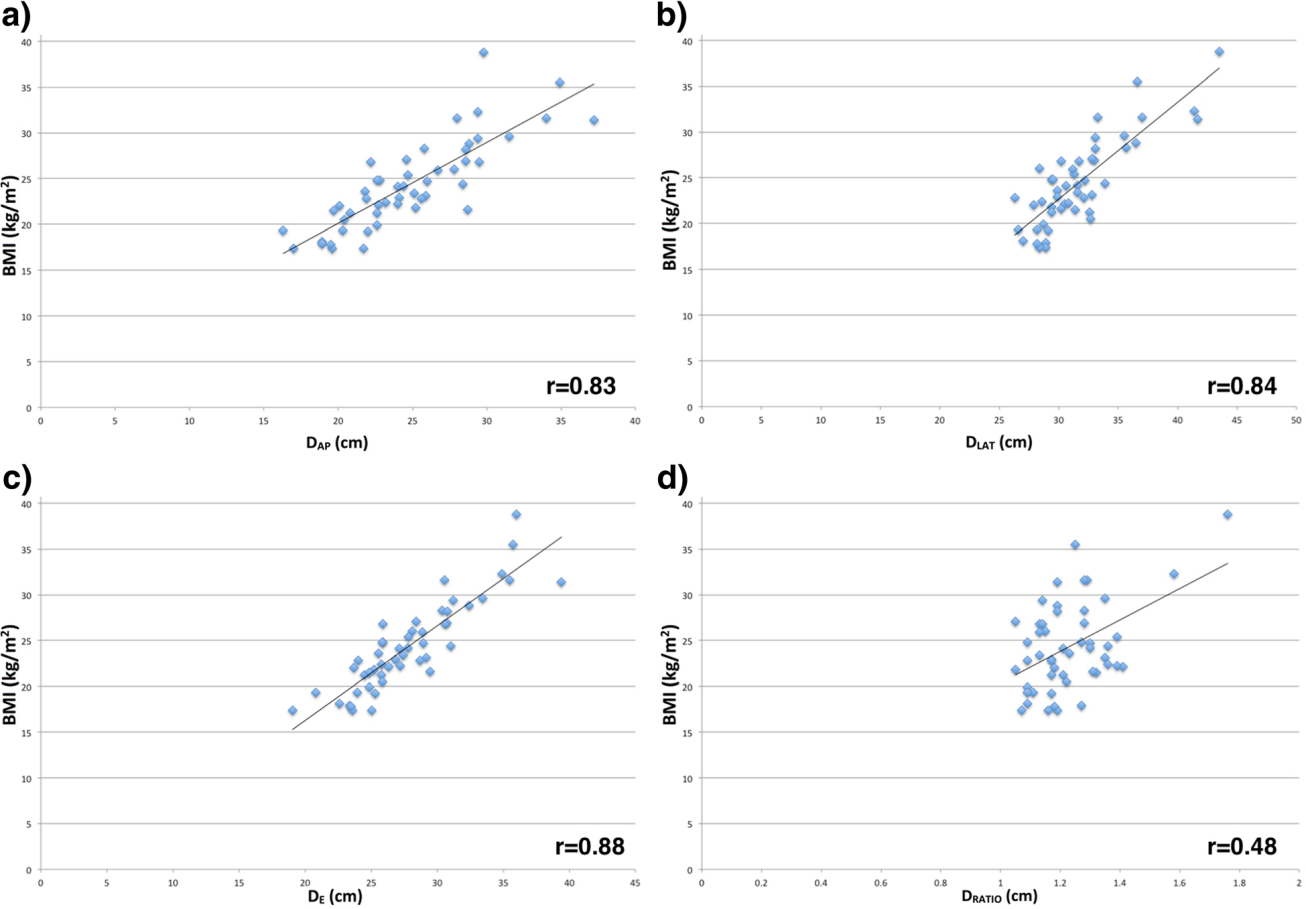
Graphs show the relationship of body mass index (BMI) to anteroposterior diameter (*D*_*AP*_) (**a**), lateral diameter (*D*_*LAT*_) (**b**), effective diameter (*D*_*E*_) (**c**) and effective diameter ratio (*D*_*RATIO*_) (**d**). Correlation coefficients were 0.83, 0.84, 0.88, and 0.48, respectively (*p* < 0.001)

The overall *D*_*AP*_, *D*_*LAT*_, *D*_*AP* + *LAT*_ and *D*_*E*_ were 24.8 ± 4.5 cm, 31.5 ± 3.9 cm, 56.3 ± 7.9 cm, and 27.8 ± 4.1 cm (mean ± SD), respectively. The effective diameter ratio (*D*_*RATIO*_) was 1.23 ± 0.13 (mean ± SD). The correlations between BMI and diameter are shown in Fig. 2. Overall, the best correlation was found with *D*_*E*_ (0.88), where correlations between mean BMI and mean body diameters were highly significant (*p* < 0.001).

Mean BMI and body diameters across BMI subgroups are shown in Table 2. Excluding *D*_*RATIO*_, the other body diameters strongly correlated with each other. The correlation coefficients (*r*) were 0.78 for *D*_*AP*_ − *D*_*LAT*_, 0.96 for *D*_*AP*_ − *D*_*E*_, and 0.92 for *D*_*LAT*_ − *D*_*E*_, (*p* < 0.001 for all). There was a moderate correlation between D_LAT_ and D_RATIO_ with a correlation coefficient of 0.62 (*p* < 0.001) and weak but statistically significant correlations between *D*_*AP*_ and *D*_*RATIO*_ (*r* = 0.34, *p* = 0.016) and *D*_*E*_ and *D*_*RATIO*_ (*r* = 0.49, *p* < 0.001). A stronger correlation was found between *D*_*AP* + *LAT*_ and *D*_*E*_ (*r* = 0.99, *p* < 0.001) than with either *D*_*AP*_ or *D*_*LAT*_ alone and *D*_*E*_ [5].

**Table 2:** Summary of body mass index category and midslice diameter measurements

	*D*_*AP*_ (cm)	*D*_*LAT*_ (cm)	*D*_*E*_ (cm)	*D*_*RATIO*_
Overall (*n* = 50)	24.77 ± 4.53	31.53 ± 3.9	27.79 ± 4.12	1.23 ± 0.13
Underweight (*n* = 6)	19.27 ± 1.52	27.42 ± 2.23	22.85 ± 2.03	1.16 ± 0.07
Normal weight (*n* = 26)	23.12 ± 2.74	30.23 ± 1.92	26.22 ± 2.16	1.22 ± 0.11
Overweight (*n* = 12)	27.35 ± 2.62	32.71 ± 2.37	29.88 ± 2.13	1.2 ± 0.1
Obese (*n* = 6)	32.22 ± 3.65	38.92 ± 3.89	35.34 ± 2.84	1.39 ± 40.23

*BMI* body mass index; data as means ± standard deviations of the mean

Underweight referred to BMI < 18.5 kg/m^2^, normal weight referred to 18.5 ≤ BMI < 25 kg/m^2^, overweight referred to 25 ≤ BMI < 30 kg/m^2^, obese referred to BMI ≥ 30 kg/m^2^

**Fig. 3:**
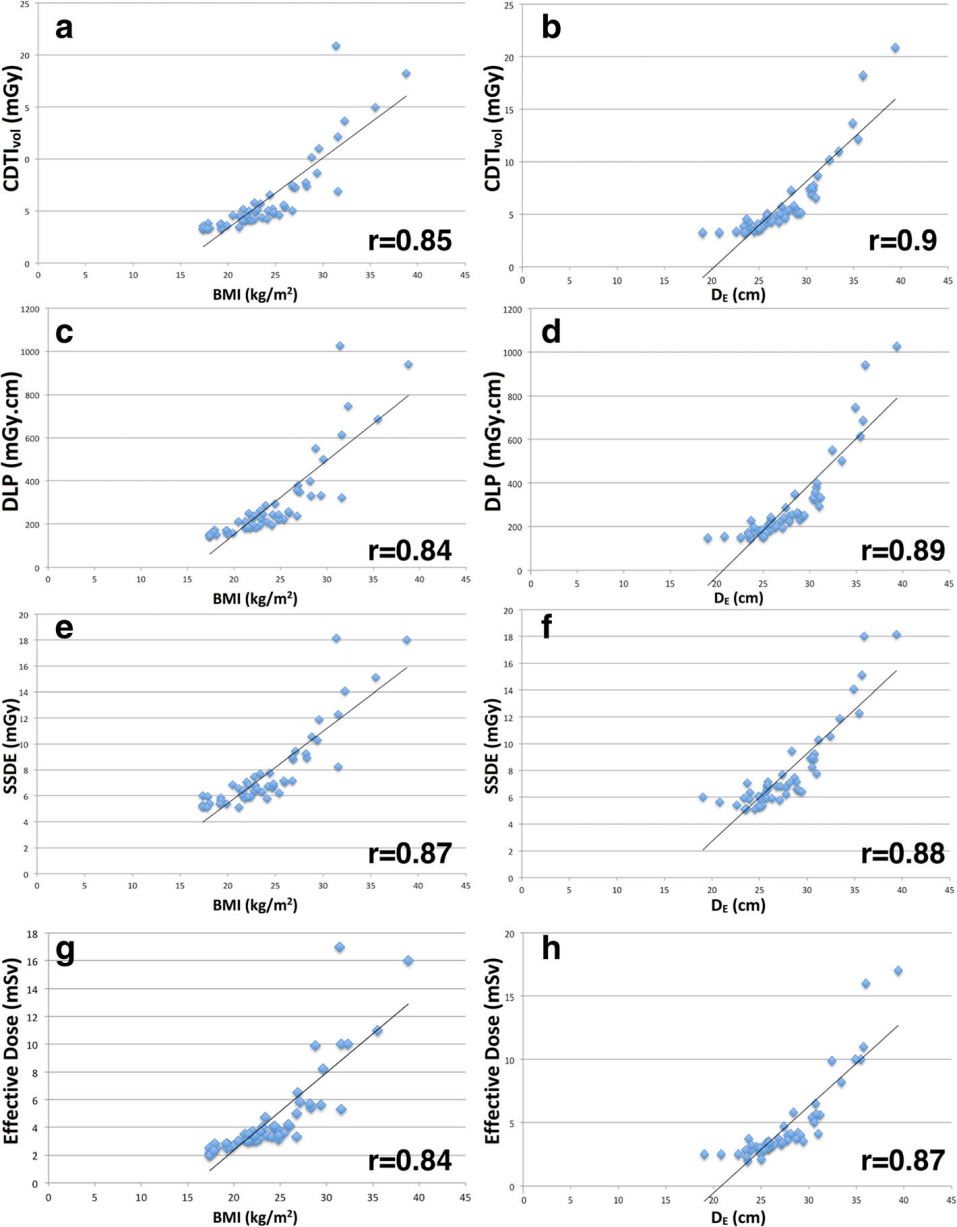
Scatterplots show: volume-computed tomography dose index (CTDI_vol_) for body mass index (BMI) (**a**) and effective diameter (**b**) with correlation *r* values of 0.85 and 0.9, respectively; dose length product (DLP) for BMI (**c**) and effective diameter (**d**) with *r* correlation values of 0.84 and 0.89, respectively; size-specific dose estimate (SSDE) for BMI (**e**) and effective diameter (**f**) with *r* correlation values of 0.87 and 0.88, respectively; effective dose for BMI (**g**) and effective diameter (**h**) with *r* correlation values of 0.84 and 0.87, respectively

Both BMI and effective body diameter correlated strongly with all the dose metrics analysed (*p* < 0.001), with *r* values ranging from 0.84 to 0.90. A weaker but significant correlation of *D*_*RATIO*_ with each dose metric was found: *r* values ranged from 0.45 to 0.48 (*p* ≤ 0.05). These data are summarised in Fig. 3.

**Fig. 4:**
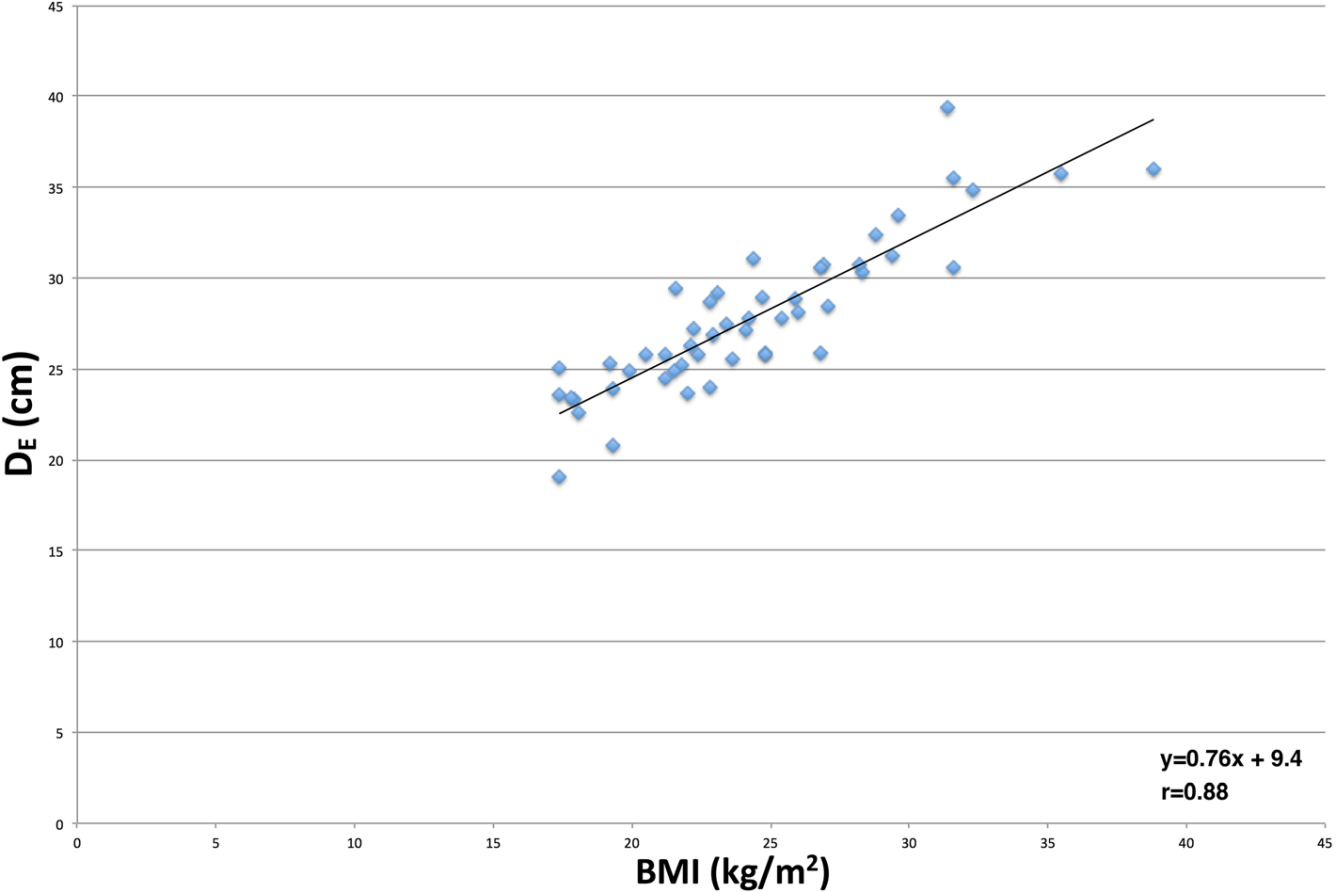
Scatterplot of body mass index (BMI) as a function of effective diameter. Linear regression trend line equation: *D*_*E*_ = 0.76(*BMI*) + 9.4

The equation of the linear regression trend-line plotting effective diameter as a function of BMI is as follows: *y* = 0.76(*x*) + 9.4 (Fig. 4). Utilising this equation, the patient’s BMI (*x*) can be used to calculate an estimate for the effective diameter (*y*), i.e. *D*_*E*_ = 0.76(BMI) + 9.4. Table 3 demonstrates a list of conversion factors to calculate SSDE from CTDI_vol_ based on the BMI of the patient.

**Table 3:** Conversion factors to convert volume-computed-tomography dose index (CTDI_vol_) to size-specific dose estimate (SSDE) based on body mass index (BMI)

BMI	*D*_*E*_	CTDI_vol_/SSDE conversion factor
15	20.8	1.73
16	21.6	1.68
17	22.3	1.63
18	23.1	1.59
19	23.8	1.54
20	24.6	1.50
21	25.4	1.46
22	26.1	1.42
23	26.9	1.38
24	27.6	1.34
25	28.4	1.31
26	29.2	1.27
27	29.9	1.23
28	30.7	1.20
29	31.4	1.17
30	32.2	1.14
31	33.0	1.10
32	33.7	1.07
33	34.5	1.04
34	35.2	1.02
35	36.0	0.99
36	36.8	0.96
37	37.5	0.93
38	38.3	0.91
39	39.0	0.88
40	39.8	0.86
41	40.6	0.84
42	41.3	0.81
43	42.1	0.79
44	42.8	0.77
45	43.6	0.75
46	44.4	0.73
47	45.1	0.71
48	45.9	0.69
49	46.6	0.67
50	47.4	0.65

*BMI* body mass index, *CTDI*_*vol*_ volume-computed-tomography dose index, *D*_*E*_ effective diameter, *SSDE* size-specific dose estimate

## Discussion

CTDI_vol_ indicates the amount of radiation delivered by the scanner for a specific CT examination, calculated on the basis of a standardised and homogenous phantom study. It is a precisely defined metrics that is displayed on the dose protocol of every CT scanner and is a measure of scanner radiation output, indicating how much radiation is directed toward the patient rather than quantifying how much radiation a patient receives [2]. Increasing use of CT and concerns regarding radiation dose from medical imaging, however, increase the need for imaging providers to facilitate accurate estimation of radiation dose to patients.

SSDE is a dose parameter that takes into consideration corrections based on the size of the patient from linear dimensions measured on the patient images. SSDE is an estimate of the mean dose to the centre of the scan volume for an object having similar attenuation characteristics as a given patient; it is not a direct measurement of dose to a specific patient [8]. This metrics provides the ability to estimate the average radiation dose to a patient in a clinical setting, albeit with 10–20% variability of the dose estimate from the actual received dose, even when patient size is taken into account [3]. The AAPM report [3] recommends that the SSDE for each patient be estimated prior to CT scan using patient size parameters to best optimise the scanning parameters to achieve the diagnostic quality CT images with the lowest necessary radiation dose.

Khawaja et al. [6] demonstrated body weight to be a more simple and convenient measure than effective diameter to estimate SSDE in paediatric patients at the time of CT scanning. They argued that measurement of body diameters in clinical practice is awkward, inconvenient, time-consuming and open to interobserver variability, particularly in the absence of a automated methods of measurement. An overview of other attempts to estimate the radiation dose in CT is presented in Table 4 [9–13]. We hypothesised that BMI, being a composite measurement derived from both weight and height, may also represent an appropriate indicator of patient size to use in lieu of effective diameter in predicting SSDE. BMI is an easily, and often routinely, obtained measurement in clinical practice. It is an objective measure with limited bias or interobserver variability during calculation [14].

**Table 4:** Examples of dose estimation on abdominal computed tomography (CT)

Monte Carlo dose estimation with patient-specific anatomical models [9]	Full-body computer model created based on the patient’s clinical CT data. Large organs individually segmented and modelled. Other organs were created by transforming an existing adult male or female full-body computer model to match the framework defined by the segmented organs, referencing the organ volume and anthropometry data in ICRP Publication 89. A Monte Carlo program (General Electric Lightspeed VCT-XTe, GE Healthcare, GE Medical Systems, Waukesha, WI, USA) was used to estimate patient-specific organ dose, from which effective dose and risks of cancer incidence were derived. Study suggests the construction of a large library of patient-specific computer models could estimate dose for any patient prior to or after a CT examination
Automated measurement of effective diameter [10]	Algorithm for estimating body-size diameter on axial CT slice implemented in Python and C#. Number of pixels whose Hounsfield unit exceeding a set threshold multiplied by the area of a single pixel to give an estimate of the area of the patient cross-section. Effective diameter computed as diameter of the circle whose area is the same as that of the cross-section. Correlation between the manual and automated measurements of effective diameter was very high
Patient size modelled as a water-equivalent diameter (*D*_W_) [11]	Water-equivalent diameter (*D*_W_), automatically extracted from axial CT images and used to model patient size and subsequently to calculate size-specific dose estimates. The extracted *D*_W_ values correlate well with effective diameter (*R*^2^ of 0.90 for abdomen and pelvis)
Dose estimation through directly using thermoluminescent dosemeters (TLDs) [12]	Thermoluminescent dosemeters (TLDs) and a Rando Alderson phantom used. Computer-simulated dose estimation based on National Radiation Protection Board Monte Carlo simulations. Directly measured dose 18% higher than computer-simulated dosimetry, suggesting underestimation by computer-simulation techniques compared with TLD measurements
Topogram-based body size indices for CT dose consideration and scan protocol optimisation [13]	Linear regression of four topographical indices for estimation of *D*_*w*_ (i) average diameter; (ii) girth (cross-section modelled as ellipse); (iii) topogram projection area; (iv) improved topogram projection area (corrected for patient miscentering and water attenuation coefficient)
Correlating body weight with diameter for radiation dose estimates [6]	Anteroposterior and lateral diameters were measured manually and through automated software. Effective diameter subsequently calculated. Overall body weight had a strong correlation with diameter

The present study assessed relevant size metrics and demonstrated a very strong correlation between effective diameter and SSDE (*r* = 0.88 or 0.87; *p* < 0.001) with BMI, indicating that BMI is an accurate alternative to effective diameter for SSDE estimation in abdominal CT.

Bias from other sources was minimised as all patients were scanned using a predefined and standardised CT abdomen and pelvis protocol on a single CT scanner by one of two radiographers. This paper demonstrates that effective diameter can be accurately estimated using an equation and patient BMI. SSDE can then be computed in a standard manner from CTDI_vol_ by using AAPM look-up tables to derive conversion factors.

We found that in patients with a wide range of body habitus measurements (BMI range, 17.4–38.8; effective diameter range, 19–39.4), BMI measurements correlated strongly with diameters. The use of BMI to calculate SSDE has been shown to be a valid alternative to the traditional methods for manual measurement of anterior-posterior and lateral-lateral dimensions using the electronic callipers available on the PACS. A recent study by Babak Alikhani et al. [15] showed that in abdominal CT, the size-dependent conversion factor (f size) closely correlated with patient BMI, indicated by the exponentially decreasing f size values with increasing BMI. The current study echoes these results with the proposition that BMI can act as a surrogate for determining effective body diameter.

The effective diameter ratio (*D*_*RATIO*_) is a new metrics proposed by Lamoureux et al. [7] as a supplement to patient-specific size parameter data and is, as yet, not validated. *D*_*RATIO*_ provides information about anatomical composition, particularly the volume of extra-abdominal adipose tissue but underestimates intra-abdominal adiposity. It proved to be a much weaker predictor of radiation dose to the patient in terms of CTDI_vol_, DLP and effective dose than either effective diameter or BMI (*p* < 0.001) in the present paper. Findings suggest that while this metrics is a good indicator of body fat distribution, it is suboptimal as a predictor of patient SSDE.

Our study has some limitations. Our sample size is relatively low (*n* = 50), with patients chosen because standardised CT imaging on this well-characterised cohort had already been performed and BMI measurements were available for all of them; CT images and data were readily available to test this hypothesis without the need to image further patients. A larger sample size may strengthen the assessment of the relationship between effective diameter and BMI. In addition, with a larger sample size, it could be possible to estimate trend lines stratified for BMI, which may lead to better estimates of diameter and hence of SSDE.

Due to the retrospective design of this study, BMI measurements only were available rather than the constituent height and weights. Statistical evaluation of the interplay of these parameters with BMI, body diameters and radiation dose may have yielded further supportive information. With the advent of automated body diameter and SSDE measurement technology, the applicability of our findings may not be relevant to the small proportion of centres that possess these technologies.

Dose optimisation is a key factor to current radiology practice, particularly for CT when the correct balance between radiation dose and image quality needs to be struck. To be useful and effective, any applied method of estimating patient radiation dose needs to be user-friendly and reproducible. The present paper indicates that patient BMI can be used to accurately estimate effective diameter, obviating the need to measure anteroposterior and lateral diameters in order to calculate SSDE at the time of CT.

## Abbreviations

AAPM: American Association of Physicists in Medicine
BMI: Body mass index
CT: Computed tomography
CTDI_vol_: Volume CT dose index
*D*_*AP*_: Maximum skin-to-skin anteroposterior diameter
*D*_*E*_: Effective diameter
*D*_*IN*_: Inner *D*_*E*_
*D*_*LAT*_: Maximum skin-to-skin lateral diameter
DLP: Dose length product
*D*_*OUT*_: Outer *D*_*E*_
*D*_*RATIO*_: Ratio between the internal and the external abdominal diameters
PACS: Picture-archiving and communication system
SD: Standard deviation
SSDE: Size-specific dose estimate
